# EUSEDcollab: a network of data from European catchments to monitor net soil erosion by water

**DOI:** 10.1038/s41597-023-02393-8

**Published:** 2023-08-04

**Authors:** Francis Matthews, Gert Verstraeten, Pasquale Borrelli, Matthias Vanmaercke, Jean Poesen, An Steegen, Aurore Degré, Belén Cárceles Rodríguez, Charles Bielders, Christine Franke, Claire Alary, David Zumr, Edouard Patault, Estela Nadal-Romero, Ewa Smolska, Feliciana Licciardello, Gilles Swerts, Hans Thodsen, Javier Casalí, Javier Eslava, Jean-Baptiste Richet, Jean-François Ouvry, Joaquim Farguell, Jolanta Święchowicz, João Pedro Nunes, Lai Ting Pak, Leonidas Liakos, Miguel A. Campo-Bescós, Mirosław Żelazny, Morgan Delaporte, Nathalie Pineux, Nathan Henin, Nejc Bezak, Noemí Lana-Renault, Ourania Tzoraki, Rafael Giménez, Tailin Li, Víctor Hugo Durán Zuazo, Vincenzo Bagarello, Vincenzo Pampalone, Vito Ferro, Xavier Úbeda, Panos Panagos

**Affiliations:** 1 European Commission, Joint Research Centre, Via Enrico Fermi, 2749, Ispra, VA 21026 Italy; 2 Earth and Environmental Sciences, KU Leuven, Celestijnenlaan 200e - box 2409, 3001 Leuven, Belgium; 3 Department of Science, Roma Tre University, Viale Guglielmo Marconi 446, 146 Roma, Italy; 4 Department of Environmental Sciences, University of Basel, Bernoullistrasse 30, 4056 Basel, Switzerland; 5 Institute of Earth and Environmental Sciences, Maria Curie-Sklodowska University (UMCS), Kra´snicka Av. 2d, Lublin, 20-718 Poland; 6 Gembloux Agro-Bio Tech, Uliège, Passage des Déportés 2, Gembloux, 5030 Belgium; 7 Natural Resources and Forestry, Instituto Andaluz de Investigación y Formación Agraria, Pesquera, Alimentaria y de la Producción Ecológica (IFAPA), Camino de Purchil s/n, Granada, 18005 Spain; 8 Earth and Life Institute - environmental sciences, UCLouvain, Croix du sud 2, Louvain-la-Neuve, 1348 Belgium; 9 Centre of Geosciences and Geoengineering, Mines Paris-PSL, 35 Rue Saint Honoré, Fontainebleau, 77305 France; 10 LGCgE, IMT Nord-Europe, 942 rue Charles Bourseul, Douai, 59508 France; 11 Department of Landscape Water Conservation, Czech Technical University in Prague, Thákurova 7, Praha 6, Prague, 16629 Czech Republic; 12 Altereo, Innovation and Digital division, 2 Av. Madeleine Bonnaud, Venelles, 13770 France; 13 Instituto Pirenaico de Ecología (IPE-CSIC), Avenida Montañana 1005, Zaragoza, 50059 Spain; 14 Faculty of Geography and Regional Studies, University of Warsaw, Krakowskie Przedmieście 30, 00-927 Warsaw, Poland; 15 Department of Agriculture, Food and Environment, University of Catania, Via Santa Sofia 100, Catania, 95123 Italy; 16 Ecoscience, Aarhus University, C.F. Møllers Allé 3, Aarhus, 8000 Denmark; 17 Department of Engineering; IS-FOOD Institute (Innovation & Sustainable Development in Food Chain), Public University of Navarre, Campus de Arrosadia, Cataluña avenue, Pamplona, Navarra 31006 Spain; 18 Division of Soils and Climatology, Department of Rural Development and Environment, Government of Navarre, González Tablas Street, 9, Pamplona, Navarra 31003 Spain; 19 AREAS, 2 Avenue Foch, 76460 Saint-Valery-en-Caux, France; 20 Geography, University of Barcelona, Montalegre 6, Barcelona, 8001 Spain; 21 Institute of Geography and Spatial Management, Jagiellonian University in Kraków, 7 Gronostajowa Str., Kraków, 30-387 Poland; 22 Soil Physics and Land Management, Wageningen University, P.O. Box 47, Wageningen, 6700 AA Netherlands; 23 cE3c - Center for Ecology, Evolution and Environmental Changes & CHANGE - Global Change and Sustainability Institute, Faculdade de Ciências da Universidade de Lisboa, Edifício C2, Piso 5, Sala 2.5.46, Campo Grande, Lisbon, 1749-016 Portugal; 24 UNISYSTEMS, Rue du Puits Romain 29, Bertrange, L-8070 Luxembourg; 25 Faculty of Civil and Geodetic Engineering, University of Ljubljana, Jamova 2, 1000 Ljubljana, Slovenia; 26 Ciencias Humanas, University of La Rioja, Luis de Ulloa 2, 26004 La Rioja, Spain; 27 Institute for Biodiversity and Ecosystem Dynamics, Universiteit van Amsterdam, Science Park 904, 1098XH Amsterdam, The Netherlands; 28 Marine Sciences Department, University of the Aegean, University hill, Mytilene, 81100 Greece; 29 Department of Agricultural, Food and Forest Sciences, University of Palermo, Viale delle Scienze, Building 4, Palermo, 90128 Italy; 30 NBFC, National Biodiversity Future Center, Palermo, 90133 Italy

**Keywords:** Hydrology, Geomorphology

## Abstract

As a network of researchers we release an open-access database (EUSEDcollab) of water discharge and suspended sediment yield time series records collected in small to medium sized catchments in Europe. EUSEDcollab is compiled to overcome the scarcity of open-access data at relevant spatial scales for studies on runoff, soil loss by water erosion and sediment delivery. Multi-source measurement data from numerous researchers and institutions were harmonised into a common time series and metadata structure. Data reuse is facilitated through accompanying metadata descriptors providing background technical information for each monitoring station setup. Across ten European countries, EUSEDcollab covers over 1600 catchment years of data from 245 catchments at event (11 catchments), daily (22 catchments) and monthly (212 catchments) temporal resolution, and is unique in its focus on small to medium catchment drainage areas (median = 43 km^2^, min = 0.04 km^2^, max = 817 km^2^) with applicability for soil erosion research. We release this database with the aim of uniting people, knowledge and data through the European Union Soil Observatory (EUSO).

## Background and Summary

Soil erosion by water and sediment delivery to river systems are gaining political importance and scientific attention for their integral role in issues spanning across the domains of soil health^1^, food security^2^, environmental pollution^3–6^, greenhouse gas offsetting^7–10^, reservoir longevity^11^, and a range of other ecosystem services^12–18^. The scientific community has responded to these priorities with a continuingly increasing number of model-based assessments, ranging across the full spectrum of spatial scales relevant to the end-user^19,20^. While model applications have dominated the scientific output, the production and sharing of empirical observations haven’t necessarily kept pace^21^. Available summarised compilations of long-term annual average rates from monitored areas have unravelled large-scale spatial trends in soil loss by water erosion and fluvial sediment yield^22–25^, but often do so with a long-term annual average temporal focus that misses the high temporal variability between soil loss events^26–28^. Quantifications of net soil loss at dynamic timescales arguably form the basis of contemporary research priorities, which include, but are not limited to: (1) understanding the variable frequency-magnitude relationships of gross and net soil loss through space and time in a changing climate, (2) understanding the influences of management practices on the dynamics and magnitude of soil loss, (3) up/down-scaling soil loss by water erosion predictions to integrate soil loss by water erosion processes into Earth system models, and (4) quantifying uncertainty on model predictions and observational data.

Given the intimate coupling between empirical observations and modelling opportunities (e.g. model development, calibration and validation), the open sharing of high resolution time series data from monitoring networks is vital to confront modern research questions^29–32^. For example, while not without criticism^33,34^, typical validation routines for spatially distributed catchment models involve the routing of overland fluxes into stream channel outlets in which an integrated comparison can be made^35–40^. The value of small monitored catchments manifests since soil erosion and sediment delivery models require an idealised ‘goldilocks’ spatial scale for such confrontations; suitably large to incorporate catchment-scale processes, but without transitioning to scales after which fluvial processes mask and confound the signal from hillslope sediment delivery^32,41^. Among the spectrum of catchment drainage areas monitored in Europe, catchments potentially matching this criteria have the lowest relative abundance^25^.

The limited open availability of suitable catchment measurements is perhaps a key underlying reason for broad critiques of model validation efforts^42^. The cascading value of available centralised monitored catchment networks (e.g. USDA-ARS) is evidenced through numerous scientific and technological advancements in soil erosion research^43–46^. In Europe, despite a relative data-richness as a continent, the absence of a multi-national network instead requires community collaborations to systematise data in a way that can unite researchers with monitoring program operators^30^. This priority is compounded by the tendency of legacy research data to become increasingly unavailable through time^47^, emphasising the general need for European data conservation efforts.

Here we present the EUropean SEDiments collaboration (EUSEDcollab) database, a multi-source platform containing over 1600 catchment years of water discharge and sediment yield time series measurements suitable for soil erosion, sediment delivery and runoff studies. The dataset originates from collaborative efforts between a network of researchers and practitioners across the community with the goal of increasing data accessibility and usability. The data collection and harmonisation campaign was undertaken in multiple phases: (1) a call of interest for participation was made to the research community, issued by the Joint Research Centre (JRC) as part of the erosion working group within the EU Soil Observatory (EUSO), (2) interested collaborators were given (meta-)data templates to compile and share time series data to a centralised data repository, and (3) following data acquisition, a harmonisation and quality checking effort was undertaken to create a standardised database from the multiple data contributors. Following this process, we provide the first data release (EUSEDcollab.v1) of a continuing collaboration and data collation campaign through the EUSO, with the broad objective of converging scientific knowledge, people and data for research and policy-related objectives in Europe^48^.

## Methods

### Data collection: scope

The initial scope of EUSEDcollab on conception was to identify and unite high value research data in predominantly agricultural landscapes across Europe. Binary conditions were not set during the data collation phase, rather holistic criteria were made to be reflected in the compiled database, such as: (1) a significant contribution of rill and inter-rill erosion to the total sediment yield among the other relevant erosion processes (i.e. landslides, gullying and river bank erosion), and (2) a small to medium spatial scale (<1000 km^2^) in which the signal of hillslope sediment delivery is reflected in the sediment yield dynamics. Following this, an inclusionary approach is taken to maximise the number of catchment datasets in the repository, allowing a user to later subset the data repository based on their needs.

### Data collection: time series and metadata structure

The monitoring of suspended sediment loads (SSL) at gauging stations requires quantifications of water discharge (Q) and suspended sediment concentration (SSC) through time. These spatial and temporal extrapolation exercises inevitably associate appreciable uncertainty with the final estimated quantity^49^. Uncertainties depends on:(1) the proficiency of Q and SSC measurement methods in capturing lateral and vertical gradients of sediment transport rate within the stream profile, (2) the timing and frequency of these measurements, and (3) the strategy used to extrapolate discrete measurements into (nearly) continuous time series. Such extrapolation is commonly undertaken using water depth-Q and Q-SSC rating curves to continuously approximate Q and SSC respectively^50,51^. In the case of SSC, surrogate approximators such as water turbidity and acoustic signals are also used to proxy changes in SSC at fine temporal resolutions based on calibrated relationships^52^. Minimising uncertainty is context-dependent based on the system dynamics^53–55^, requiring a strategic SSC sampling technique using random, calendar-based, or flow-proportional sampling schemes. Particularly at small spatial scales, a high number of SSC samples over time and using flow-proportional sampling regimes typically associates lower uncertainties with time-integrated sediment load approximations^49^.

Given the method dependency of SSL quantifications, we invited data contributors to add descriptive metadata properties of the water discharge and SSC measurement methods to provide users with background context for each timeseries (Table 1). Additionally, for the popular case in which a sediment rating curve was used for the extrapolation of SSC, we invited the contributing scientists to include the original data in order for a user to reproduce the time series of SSL.

**Table 1:** The standardised metadata template issued to the collaborating data producers of EUSEDcollab in the data collection campaign.

Variable	Descriptor	Type	% populated in database
Catchment ID	Catchment property	Assigned (num)	100
Catchment name	Catchment property	Open (text)	100
Latitude (4 decimal places)	Catchment property	Open (num)	100
Longitude (4 decimal places)	Catchment property	Open (num)	100
Country	Data record property	Assigned (text)	100
Drainage area (ha)	Catchment property	Open (num)	98
Stream type	Catchment property	Cat (text)	100
Data type	Data record property	Assigned (text)	100
Land use: % agriculture	Catchment property	Open (num)	52
Land use: % pasture	Catchment property	Open (num)	10
Land use: % shrubland	Catchment property	Open (num)	9
Land use: % forest	Catchment property	Open (num)	49
Land use: % built-up	Catchment property	Open (num)	47
Land use: % other (specify in optional column)	Catchment property	Open (num)	4
Measurement start date (DD/MM/YYYY)	Data record property	Open (date)	100
Measurement end date (DD/MM/YYYY)	Data record property	Open (date)	100
Major data gaps (DD/MM/YYYY - DD/MM/YYYY, ….)	Data record property	Open (date)	4
Average number of station checks per month	Quality control property	Open (num)	95
Water depth measurement method	Water discharge	Cat (text)	93
Number of water depth measurements per day	Water discharge	Open (num)	92
Stage-discharge conversion method	Water discharge	Cat (text)	94
Includes precipitation	Data record property	Assigned (bool)	13 (n = 32)
Suspended sediment sampling method	Sediment (direct)	Cat (text)	100
Sampling frequency (n per day or n Q-SSC pairs)	Sediment (direct)	Open (num)	81
Sampling regime	Sediment (direct)	Cat (text)	96
Suspended sediment extrapolation method	Sediment (indirect)	Cat (text)	96
Estimated bedload contribution (eg < x %)	Sediment	Open (num)	1
Includes sediment rating curve data	Data record property	Assigned (bool)	2 (n = 7)
Relevant references with full details	Literature	Open (text)	100
Contact name	Dataset inquiries	Open (text)	100
Contact email	Dataset inquiries	Open (text)	100

Each time series of water discharge and sediment yield has an accompanying metadata file to allow the filtering based on method or catchment attributes and provide the user with relevant contextual information (e.g. method descriptors and published work). Metadata identifiers were open or categorical for the data producer, or otherwise assigned during the database harmonisation process. The ‘% populated’ column refers to the % completeness of each metadata field for the entire collected database. For Boolean variables, the % populated column gives the database % with an accompanying count of the cases with a true value (i.e. containing precipitation or sediment rating curve data).

Each data entry has a standardised format with a column for the datetime, water discharge (Q: volume time^−1^), suspended sediment concentration (SSC: mass volume^−1^) and the derived suspended sediment load (SSL: mass time^−1^) accompanied by the relevant units. A metadata file accompanies each catchment entry to allow data contextualisation using open or categorical properties (Table 1). Input fields predominantly define descriptive properties of the catchment (e.g. monitoring station location, catchment drainage area and land cover), the data record (e.g. temporal extent) and the methods used to measure and quantify the water discharge and sediment yield. Land cover information is included as a metadata field since it gives the opportunity for data contributors to add and qualify primary descriptive catchment properties with more localised detail than is possible with auxiliary large-scale landcover datasets.

At minimum, each catchment entry contains a Q and SSL timeseries with a metadata file providing the geographic coordinates of the monitoring station location. However, for the majority of catchment entries the population of each metadata field within EUSEDcollab is relatively high (Table 1). Where possible, we also include: (1) precipitation time series data and rain gauge location information, (2) accompanying literature references from relevant publications for each dataset, and (3) a readme file to give expert-based contextual information to the end-user and qualify any necessary considerations within the time series data. For catchments without an associated English language publication, the submission of this file is emphasised in order to supplement the metadata with sufficient background information.

## Data Records

**Fig. 1:**
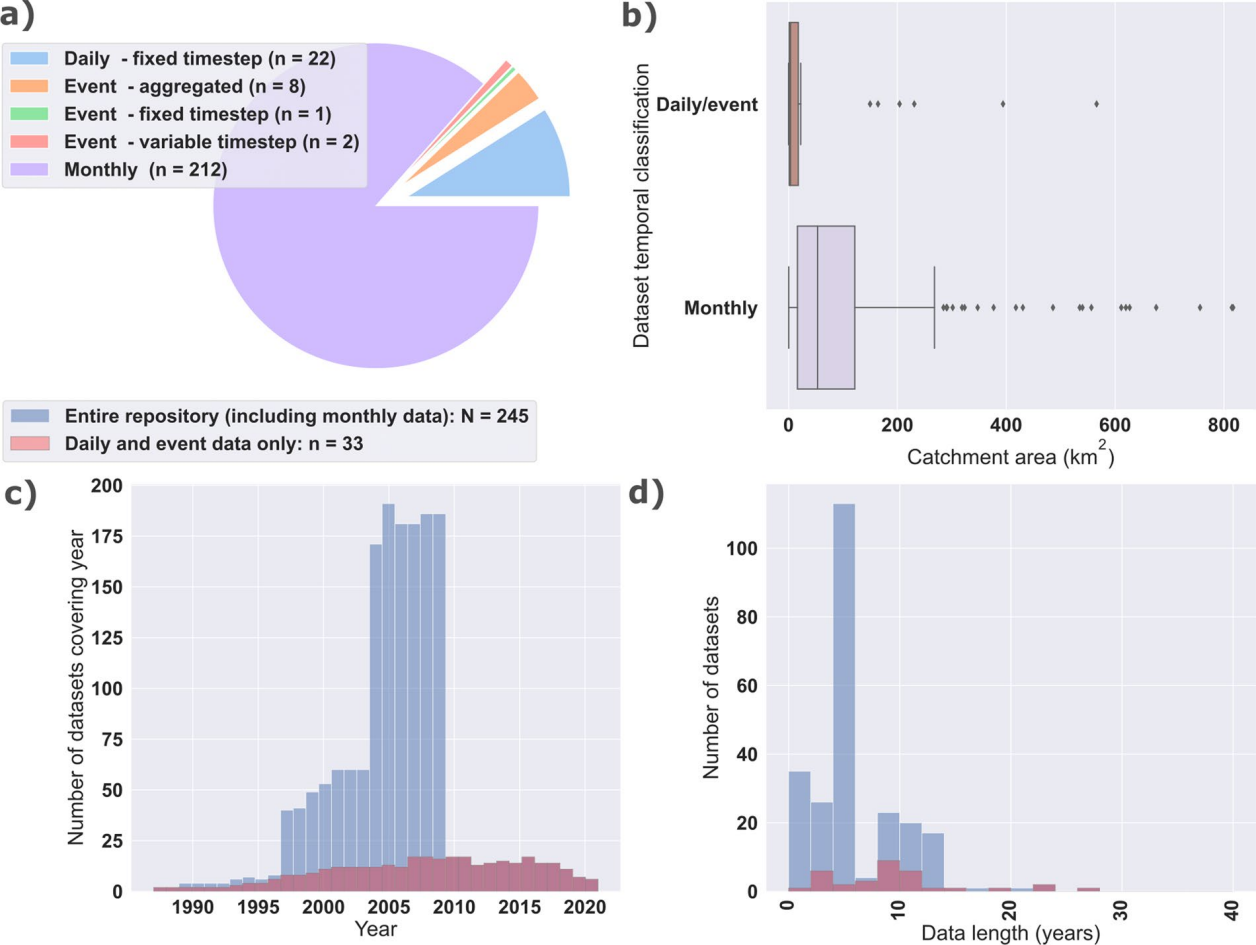
A statistical overview of the EUSEDcollab database. Catchment records are categorised into ‘Monthly’ data, with quantifications of sediment yield per month, and ‘Daily/event’ data, including all other data time structures with daily timesteps or time-distributed and time aggregated event data. The plotted overviews include: (**a**) the number of datasets belonging to each classified time-structure type, (**b**) the distribution of measurement record lengths within the database, (**c**) the number of datasets with coverage in each year, and (**d**) boxplot distributions of catchment drainage areas within the dataset for monthly and daily/event time series records.

The EUSEDcollab repository contains 245 catchments with time series of Q and SSL (Tables 2–4). We include a further seven catchment records with full Q time series and intermittent SSC measurements for a user to define their own extrapolation method, since no prior extrapolation was completed in these cases. These records are not considered in the subsequent summary but are included in the data release with accompanying metadata files. The combined dataset covers over 1600 catchment years of water discharge and suspended sediment load records. Based on time-structure, this repository is divided into 22 daily data records, 212 monthly records, 1 event record with a fixed timestep, 2 event records with variable timesteps, and 8 event records with event aggregations (Fig. 1). A large addition of data was made available from monitored Danish catchments^56^, which have a comparatively lower temporal resolution (monthly) than other individual or small collections of monitored catchments (Tables 2–4).

**Table 2:** An overview of database entries with individual event measurements and their respective assigned IDs and classified temporal structure.

Catchment ID	Catchment name	Country	Start date	End date	Drainage area (ha)	Data type	Literature references
1	Chastre-P1	BE	2012-10-05	2021-07-13	4	Event data - aggregated	^65,66^
2	Chastre-P2	BE	2013-07-27	2021-12-24	85	Event data - aggregated	^65,66^
3	Chastre-P3	BE	2017-12-31	2021-12-24	112	Event data - aggregated	^65,66^
4	Chastre-P4	BE	2013-06-20	2021-06-03	356	Event data - aggregated	^65,66^
5	Ganspoel	BE	1997-03-01	1999-03-01	117	Event data - variable timestep	^35,67–69^
6	Kinderveld	BE	1996-07-01	1999-11-01	250	Event data - variable timestep	^35,67–69^
8	BRVL	FR	2007-09-01	2018-08-31	1045	Event data - aggregated	^70–74^
9	FDTL	FR	2011-11-01	2018-08-31	145	Event data - aggregated	^70–74^
10	Pommeroye	FR	2016-03-31	2018-02-01	54	Event data - fixed timestep	^75^
16	Cannata 2	IT	1996-10-08	2006-03-18	130	Event data - aggregated	^76^
17	SPA1	IT	1997-12-24	2020-04-27	4	Event data - aggregated	^77,78^

The associated timeseries data contains either a variable or fixed sub-event timestep, or the data is aggregated per event. The ‘Literature references’ column gives the corresponding studies on the catchment undertaken before the data submission phase.

**Table 3:** An overview of database entries with a daily timestep and their respective assigned IDs.

Catchment ID	Catchment name	Country	Start date	End date	Drainage area (ha)	Data type	Literature references
7	Nučice	CZ	2014-01-13	2021-07-27	53	Daily data - fixed timestep	^79–81^
11	Airport Rasina	GR	2000-01-01	2011-09-30		Daily data - fixed timestep	^82,83^
12	Kelefina Kladas	GR	2000-01-01	2011-09-30	14980	Daily data - fixed timestep	^82,83^
13	Koumousta Rasina	GR	2000-01-01	2011-09-30		Daily data - fixed timestep	^82,83^
14	Vasaras	GR	2000-01-01	2011-09-30	16440	Daily data - fixed timestep	^82,83^
15	Vivari	GR	2000-01-01	2011-09-30	39410	Daily data - fixed timestep	^82,83^
18	Szeszupa 1- Lopuchowo	PL	1987-01-11	2010-10-31	1420	Daily data - fixed timestep	^84–87^
19	Szeszupa-Udziejek	PL	1987-11-01	1999-10-31	1580	Daily data - fixed timestep	^84–87^
20	Stara Rzeka (Gróbka)	PL	1993-11-01	2019-12-31	2240	Daily data - fixed timestep	^88,89^
21	Macieira	PT	2010-11-09	2014-09-18	94	Daily data - fixed timestep	^90,91^
22	Odeaxere	PT	2001-12-01	2005-12-31	1887	Daily data - fixed timestep	^92^
23	Mislinja	SI	2016-09-29	2019-12-31	23100	Daily data - fixed timestep	^93^
24	Rižana	SI	2017-01-01	2019-12-31	20400	Daily data - fixed timestep	^93^
25	Sora_t	SI	2016-01-01	2019-12-31	56600	Daily data - fixed timestep	^93^
26	Arnas	ES	1999-10-01	2008-09-30	284	Daily data - fixed timestep	^93^
27	El Salado	ES	2005-10-03	2021-06-05	670	Daily data - fixed timestep	^94^
28	La Tejeria	ES	2007-10-01	2016-09-30	169	Daily data - fixed timestep	^95,96^
29	Landazuria	ES	2007-10-01	2016-09-30	480	Daily data - fixed timestep	^95,97^
30	Latxaga	ES	2007-10-01	2016-09-30	207	Daily data - fixed timestep	^96,98,99^
31	Oskotz forestal	ES	2007-10-01	2016-09-30	434	Daily data - fixed timestep	^95,100^
32	Oskotz principal	ES	2007-10-01	2016-09-30	1688	Daily data - fixed timestep	^95,100^
33	Vernega	ES	1994-10-01	2012-09-30	257	Daily data - fixed timestep	^101,102^

The ‘Literature references’ column gives the corresponding studies on the catchment undertaken before the data submission phase.

**Table 4:** An overview of database entries with monthly data or only daily discharge and sediment rating curve data.

Catchment ID	Catchment name	Country	Start date	End date	Drainage area (ha)	Data type	Literature references
34–245	Denmark - multiple	DK	1997-01-01	2009-12-01	38–81682	Monthly data	^56^
246–252	Slovenia - multiple	SI	1967-01-01	2011-12-31	9200–59300	Q and rating curve data only	^93^

‘Q and rating curve data only’ signifies that the dataset contains continuous water discharge records and matching Q-SSC pairs, but no extrapolation has been performed. The ‘Literature references’ column gives the corresponding studies on the catchment undertaken before the data submission phase.

**Fig. 2:**
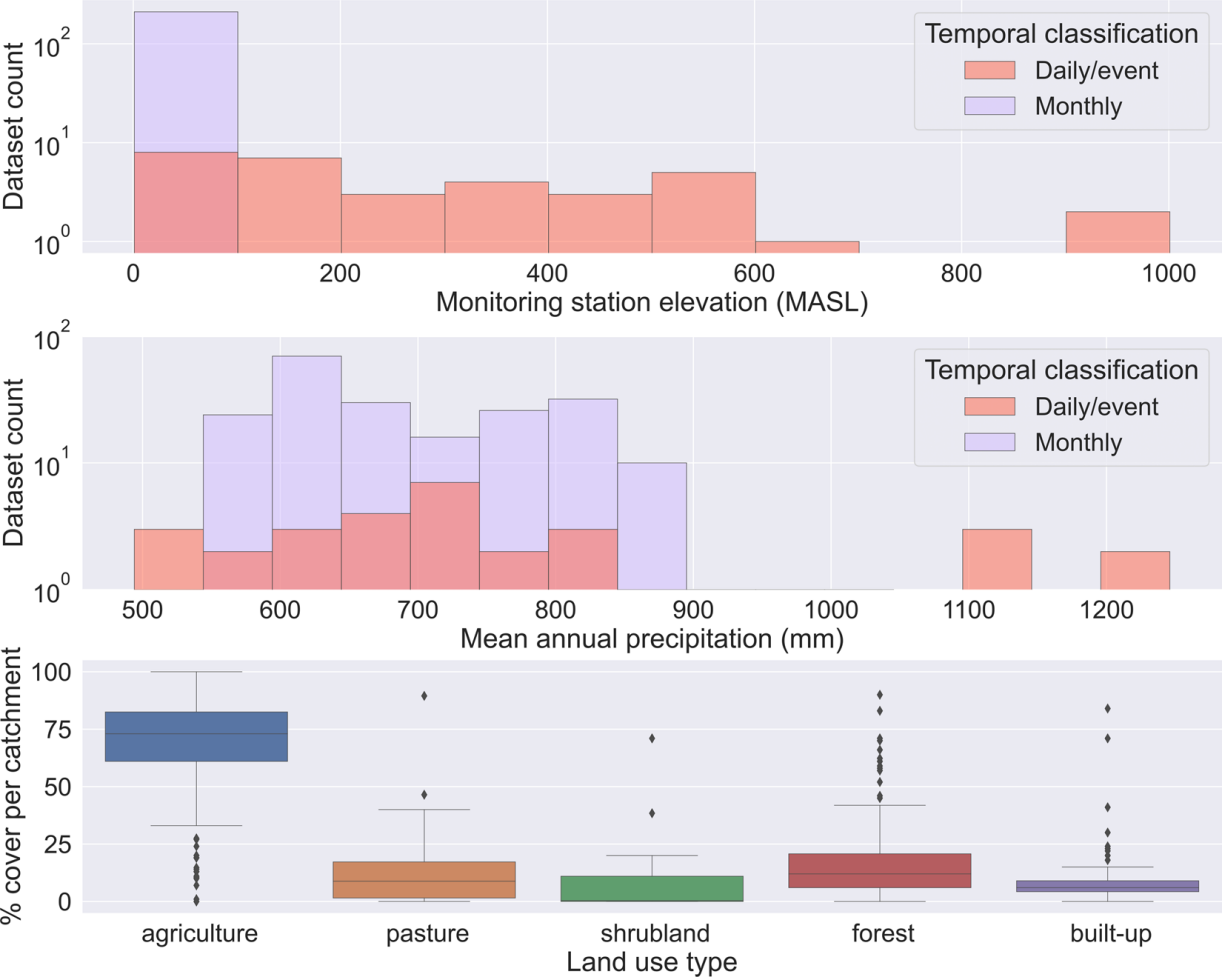
Histogram charts of the elevation (**a**) and mean annual precipitation in mm (**b**) of the monitoring stations included in EUSEDcollab. The distribution of the % cover of each land use type within the database is given for catchments with metadata inputs (**c**). Elevation is extracted from the SRTM global digital elevation layer and total annual average precipitation from Worldclim^103^.

The distribution of catchment drainage areas (median = 43 km^2^, min = 0.04 km^2^, max = 817 km^2^) included in EUSEDcollab reflects the overall focus on small to medium monitored catchment areas relevant for soil erosion and hydrological research (Fig. 1). These catchments distribute across a range of elevation settings and climatic regions but contain an overall dominance of agricultural land uses (Fig. 2). Excluding catchment entries with monthly resolution data, this median drainage area reduces to 3.6 km^2^ (min = 0.04 km^2^, max = 566 km^2^). The mean measurement length of all records is 6.7 years and 9.7 years for only high temporal resolution (excluding monthly data) records. These years of data coverage are predominantly concentrated from the year 1995 onwards (Fig. 1).

**Fig. 3:**
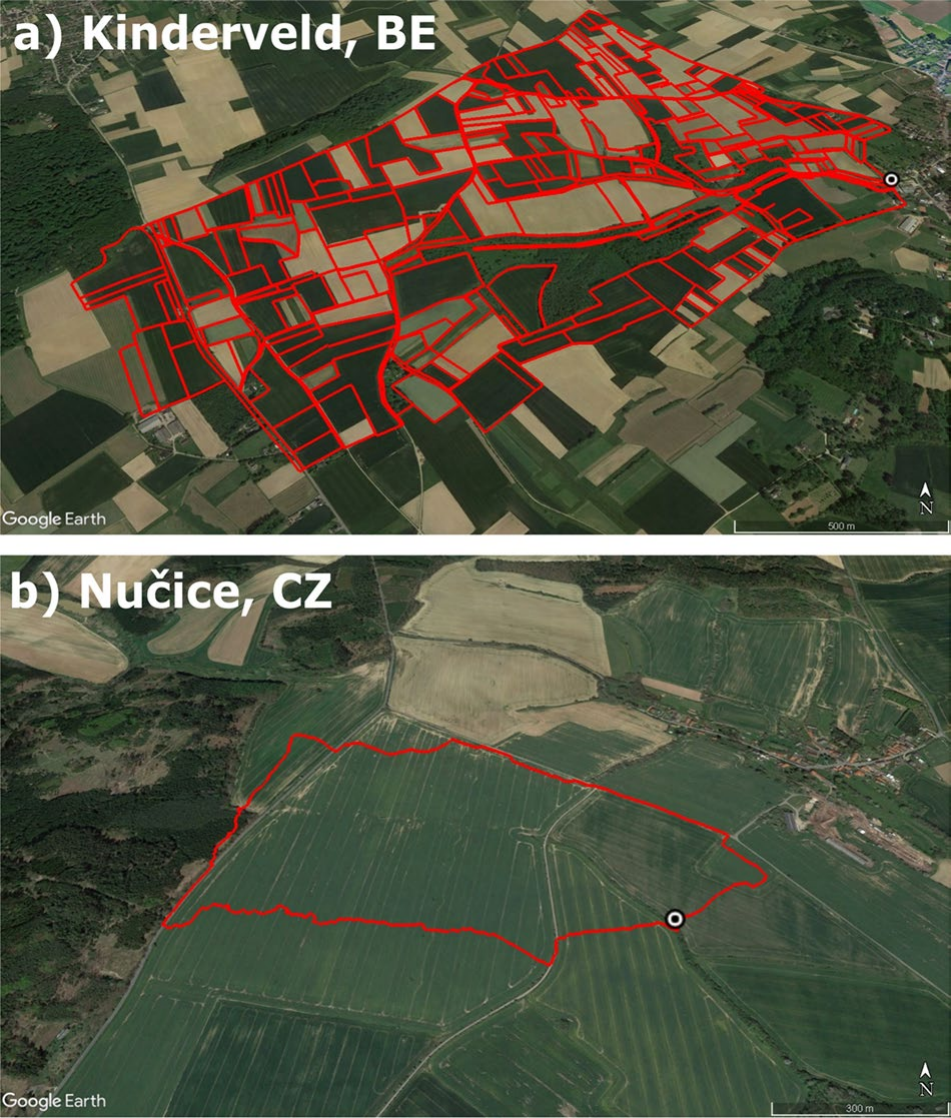
Google Earth satellite image examples of monitored catchments in EUSEDcollab with included catchment boundary polygons: (**a**) Kinderveld, BE (including parcel boundary information), and (**b**) Nučice, CZ. The point markers represent the registered monitoring locations in EUSEDcollab.

**Fig. 4:**
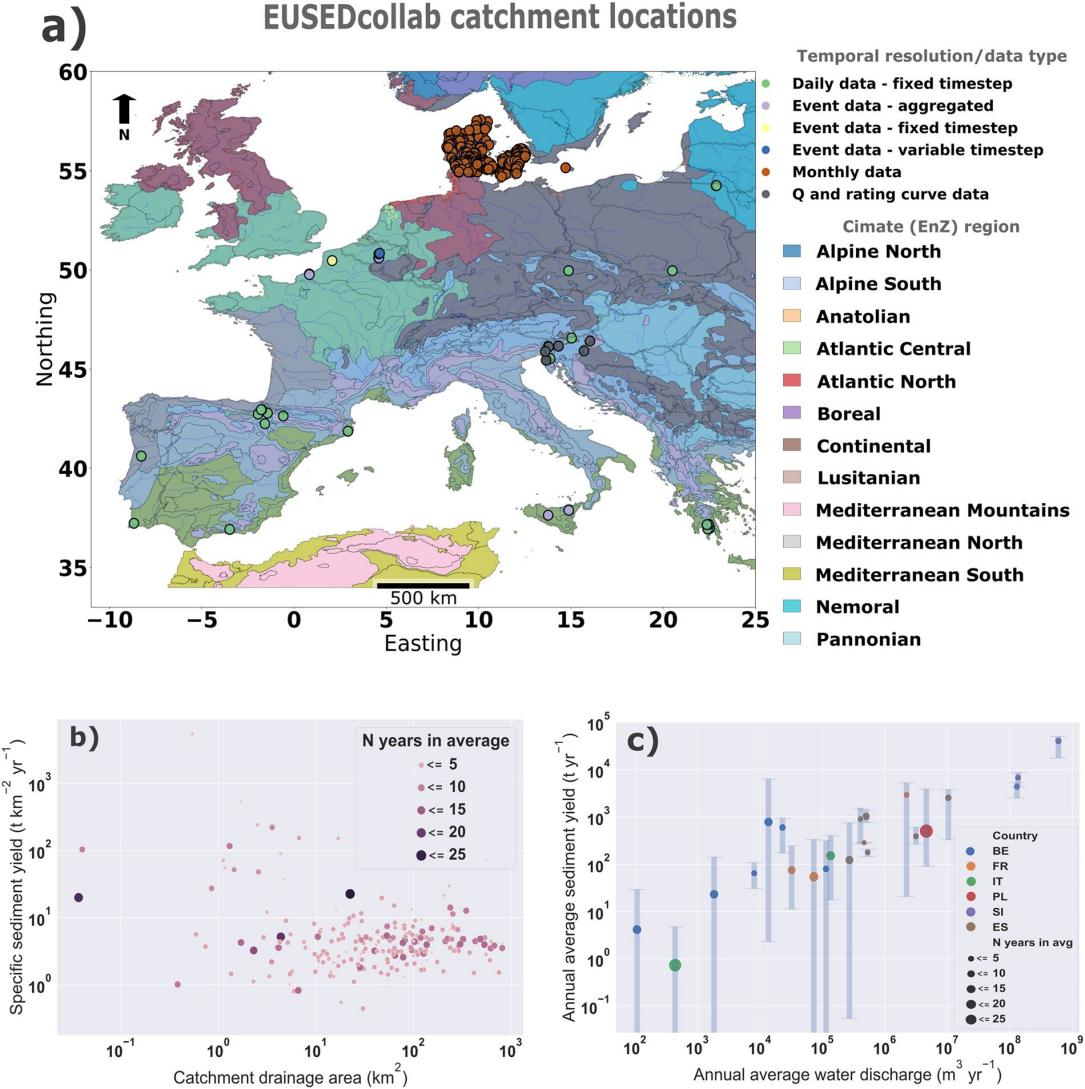
Top: A geographical overview of EUSEDcollab.v1 data entries per climate (EnZ) region in Europe^104^ (**a**). Bottom: summary-level empirical relationships found within the database entries, showing a) the relationship between catchment area (km^2^) and specific sediment yield (t km^2^ yr^−1^), and (**b**) the relationship between mean annual discharge (m^3^ yr^−1^) and the mean annual sediment yield (t yr^−1^) for all high temporal resolution datasets (excluding monthly data). The error bars show the variation of the annual sediment yield values around the mean annual average.

Of the total repository, 32 catchment entries contain additional time series measurements of precipitation depth at varying temporal resolutions for their respective location depending on the method employed. This precipitation file gives additional information on the rain gauge type and spatial coordinates. A total of 228 catchments have catchment boundary polygons added as additional information by the data provider (Fig. 3). Some monitored catchments, such as Kinderveld and Ganspoel^35^, contain additional geospatial information on land use as well as erosion surveys. In these cases we include the data in the original format and structure in which it was made available by the data producers. A full overview of all catchment locations is given in Fig. (4).

## Technical Validation

Technical validation of each original record is done in a decentralised manor by the data producer. The multi-source nature of EUSEDcollab means that measurements of Q and SSL measurements were acquired with varying apparatus set-ups, temporal structures and post-processing methods (Tables 2–4). Acknowledging varying degrees of data heterogeneity requires end-users to make a judgement on the inter-comparability of catchment records for a particular use-case, based on differing measuring extents, sampling resolutions and uncertainty sources. As a data integration and harmonisation exercise, we aimed to facilitate this user-side assessment by providing necessary metadata properties, namely: (1) water discharge method descriptors, (2) sediment flux measurement and quantification methods, and (3) quality control properties describing the frequency of monitoring station checks, (4) literature references, and (5) dataset contact information (Table 1).

### Data evaluation: quality and completeness assessment

**Fig. 5:**
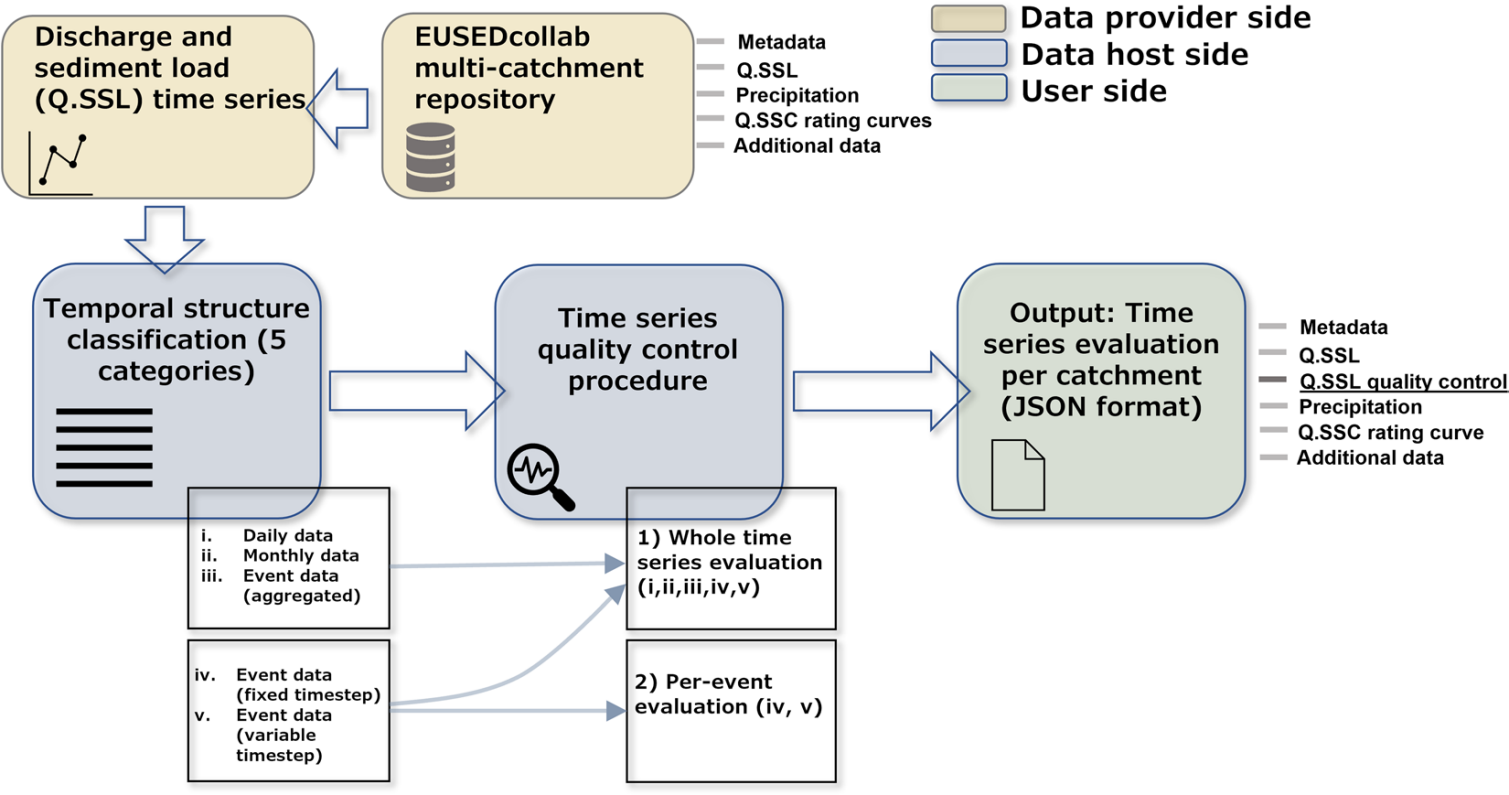
An overview of the data quality control procedure to include an evaluation of missing data entries within each time series record. A modified evaluation is made according to the time series structure of each data record. The output of the quality control procedure provides an accompanying JSON file for each data entry within EUSEDcollab.

To give a centralised assessment of the completeness and consistency of each submitted time series record, a ready-to-use evaluation was made of missing data inputs (Fig. 5). For example, missing inputs could be due to temporary technical issues, incomplete measurements or periodic discontinuation. Depending on the use-case, missing data may limit the applicability of a catchment dataset to a certain task and therefore may be useful for a user to know a priori.

The compiled time series entries in EUSEDcollab contain continuous measurements (e.g. with a daily or monthly timestep) in perennial streams or episodic measurements (e.g. time-aggregated or time-distributed events) in discontinuous streams. Based on these structural data characteristics, adapted evaluation routines were used to summarise data presence/absence through time (Fig. 5). Each time series entry is initially classified into one of five structures: (1) daily data series with a fixed timestep, (2) monthly data series with a fixed timestep, (3) event data with a fixed timestep within each event, (4) event data with a variable timestep within each event, or (5) event data that is temporally-aggregated per event. Thereafter, evaluations of each time series are made to give the total % completeness of the instances for both Q and SSL. For data containing fine-resolution measurements during episodic events, within-event evaluations are additionally generated to quantify the completeness of each individual event making up the entire time series (Fig. 5). A full description of each evaluation parameter is given in S.(1) for each classified time series structure.

## Usage Notes

### Data opportunities

**Fig. 6:**
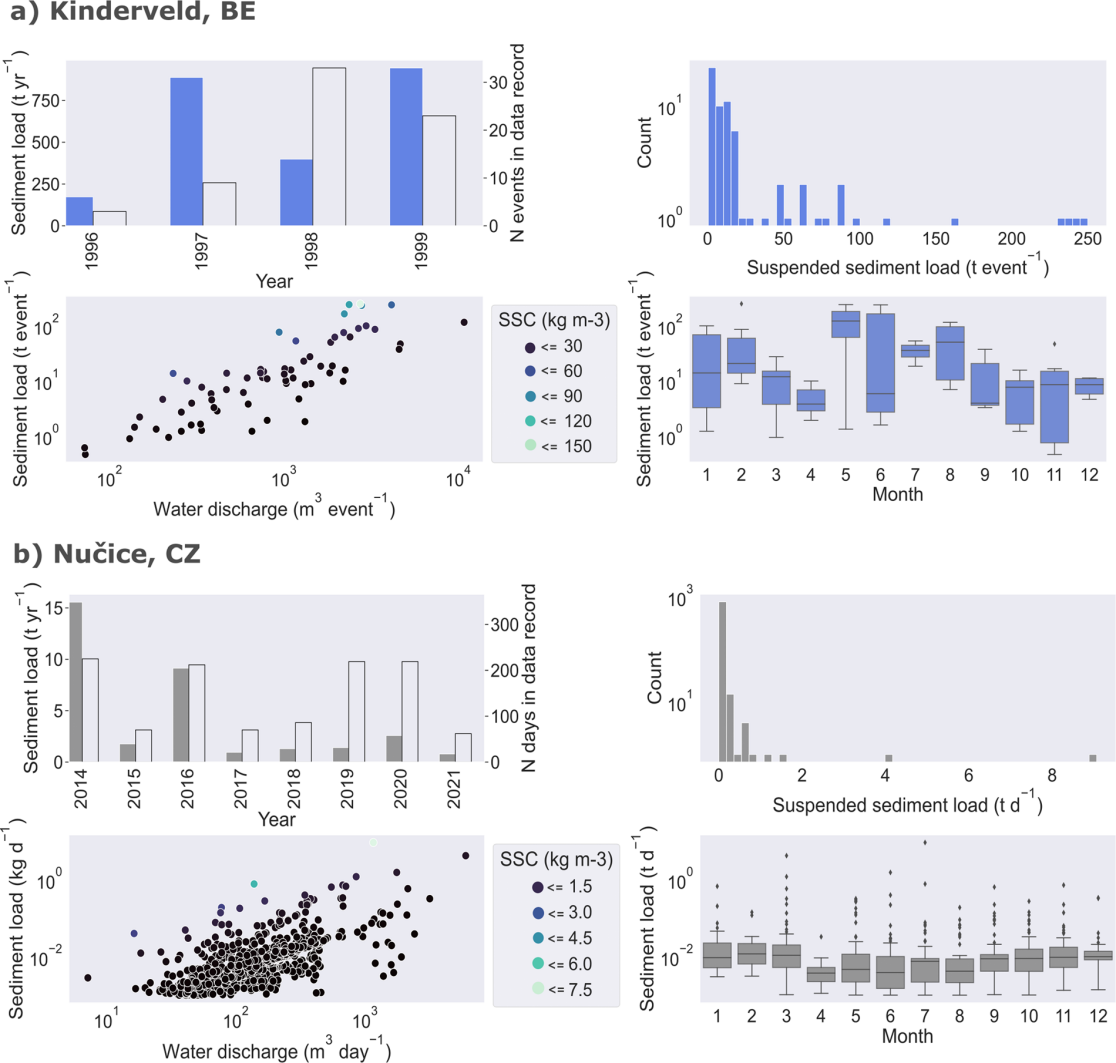
Example syntheses of time series data from the Kinderveld catchment, BE (250 ha) and the Nučice catchment, CZ (53 ha) in the EUSEDcollab repository. Note that the data is not area-normalised and the data from the Kinderveld catchment (**a**) is presented in tonnes per aggregated event, while the Nučice catchment (**b**) is made available and presented in tonnes per day. Additionally, it is important to consider the following contextual factors: (i) The Nučice measurements include periods with baseflow carrying sediments, whereas in the Kinderveld, only runoff events are included. This difference in sediment sources (rill and interrill, bank erosion and gullying) between the two catchments, explained in the related literature (Tables 2, 3), may contribute to variations in the observed values. (ii) In Nučice, the low number of days in the data record for specific years (e.g., 2015, 2017, 2018, 2021) is due to exceptionally dry years when the discharge was zero or very low, limiting the availability of sediment data.

EUSEDcollab is the first database of its kind in Europe, intended as a resource for a non-exhaustive range of applications relating to runoff, soil loss by water erosion and sediment delivery research at singular or multiple sites. These opportunities can include a range of research domains seeking to understand the system dynamics of catchment-scale runoff, erosion and sediment fluxes (Figs. 4, 6). These may include modelled and analytical developments in frequency-intensity relationships^26,27,57,58^, spatial and temporal scale-effects^25,59–61^, or internal (e.g. topography, geology, soil characteristics), external (e.g. meteorological conditions) and anthropogenic (e.g. land use and land cover) drivers of sediment variability^62^.

By uniting data from across a European scientific network, we aim to: (1) release an open-access data resource hosted on the European Soil Data Centre (ESDAC) with the goal of continued database growth in a standardised manor, (2) mitigate data loss from discontinued research projects, (3) build a repository upon which a broad range of analytical and modelling methods can be built to advance scientific knowledge, and (4) allow cross-domain intercomparisons to assess the generalisation of empirical relationships and model prediction systems.

### Data limitations

Data users are advised to consider the applicability of each utilised dataset for their application. These considerations range from the spatial scale (drainage area) of the catchment in its context-dependent environmental setting, to the temporal detail and measurement-richness underlying the dataset. The data quality evaluation gives additional relevant information on the time series completeness in order for initial evaluations to be made (Fig. 5).

The EUSEDcollab.v1 repository has a significant spatial bias in its coverage due to a large number of data additions from small to medium sized catchments from a national monitoring campaign in Denmark^56^. These data have evidenced usage in erosion modelling^36^ but may not meet the requirements of certain high temporal resolution research applications due to infrequent underlying suspended sediment sampling. We envisage that continued catchment data inputs from national monitoring campaigns fitting the motivations of EUSEDcollab will improve the overall spatial coverage and reduce this spatial bias.

### Data platform and continued community contributions

The EUSEDcollab repository is openly accessible via the European Soil Data Centre^63^ (ESDAC) platform (https://esdac.jrc.ec.europa.eu/content/EUSEDcollab) and Figshare^64^. All files are provided in .csv format in their relevant folders and are identifiable based on the assigned ID listed in the overview file (Catchment_ID_assignment.csv). In the case of database-wide applications, users are requested to cite this article as the reference for the entire repository. In cases of individual catchment applications, users should refer to the reference studies for each catchment provided in the metadata and summarised in Tables 2–4.

EUSEDcollab.v1 is intended as the first version of a continued effort to gather and platform data through collaborative efforts from across the community. Future data collection efforts will seek to extend the size and scope of the repository through including a wider diversity of catchment types (e.g. pristine forests, badlands etc.) across a wider range of elevation settings.

Further contributions can be made to the database by downloading and completing the data and meta-data template files available in the ESDAC data portal (https://esdac.jrc.ec.europa.eu/content/EUSEDcollab). Data submissions can be included in future data releases by contacting the listed data manager through the contact details listed in the ESDAC data portal.

## Data Availability

All code can be found at: https://github.com/matfran/EUSEDcollab.git. We include the R language code to perform the quality control procedure on each time series entry to produce the JSON time series evaluation files for each record. Additionally, a Python language Jupyter notebook is included to demonstrate simple operations that can be undertaken using the database, such as reading and filtering the database, calculating metadata statistics and importing specific time series for analysis.

## References

[1] Montanarella L, Panagos P (2021). The relevance of sustainable soil management within the European Green Deal. Land use policy.

[2] Alewell C (2020). Global phosphorus shortage will be aggravated by soil erosion. Nature Communications 2020 11:1.

[3] Panagos P, Jiskra M, Borrelli P, Liakos L, Ballabio C (2021). Mercury in European topsoils: Anthropogenic sources, stocks and fluxes. Environ Res.

[4] Ulén B, Bechmann M, Fölster J, Jarvie HP, Tunney H (2007). Agriculture as a phosphorus source for eutrophication in the north-west European countries, Norway, Sweden, United Kingdom and Ireland: a review. Soil Use Manag.

[5] Mullan D, Vandaele K, Boardman J, Meneely J, Crossley LH (2016). Modelling the effectiveness of grass buffer strips in managing muddy floods under a changing climate. Geomorphology.

[6] Boardman J, Vandaele K, Evans R, Foster IDL (2019). Off-site impacts of soil erosion and runoff: Why connectivity is more important than erosion rates. Soil Use Manag.

[7] Chappell A, Baldock J, Sanderman J (2015). The global significance of omitting soil erosion from soil organic carbon cycling schemes. Nature Climate Change 2015 6:2.

[8] Kuhn NJ, Hoffmann T, Schwanghart W, Dotterweich M (2009). Agricultural soil erosion and global carbon cycle: Controversy over?. Earth Surf Process Landf.

[9] Borrelli P (2016). Effect of Good Agricultural and Environmental Conditions on erosion and soil organic carbon balance: A national case study. Land use policy.

[10] Borrelli P (2018). A step towards a holistic assessment of soil degradation in Europe: Coupling on-site erosion with sediment transfer and carbon fluxes. Environ Res.

[11] Graf WL, Wohl E, Sinha T, Sabo JL (2010). Sedimentation and sustainability of western American reservoirs. Water Resour Res.

[12] Borrelli, P. *et al*. Policy implications of multiple concurrent soil erosion processes in European farmland. *Nature Sustainability 2022* 1–10, 10.1038/s41893-022-00988-4 (2022).

[13] Borrelli P, Panagos P (2020). An indicator to reflect the mitigating effect of Common Agricultural Policy on soil erosion. Land use policy.

[14] Panagos, P. & Katsoyiannis, A. Soil erosion modelling: The new challenges as the result of policy developments in Europe. *Environmental Research* vol. 172, 470–474, 10.1016/j.envres.2019.02.043 (2019). 10.1016/j.envres.2019.02.043 30844572

[15] Panagos P (2021). Projections of soil loss by water erosion in Europe by 2050. Environ Sci Policy.

[16] Quinton JN, Govers G, Van Oost K, Bardgett RD (2010). The impact of agricultural soil erosion on biogeochemical cycling. Nature Geoscience 2010 3:5.

[17] Lal R (2014). Soil conservation and ecosystem services. International Soil and Water Conservation Research.

[18] Issaka, S. & Ashraf, M. A. Impact of soil erosion and degradation on water quality: a review. **1**, 1–11, 10.1080/24749508.2017.1301053 (2017).

[19] Tang T (2019). Bridging global, basin and local-scale water quality modeling towards enhancing water quality management worldwide. Curr Opin Environ Sustain.

[20] Borrelli P (2021). Soil erosion modelling: A global review and statistical analysis. Science of The Total Environment.

[21] Vörösmarty, C. *et al*. Global water data: A newly endangered species. *Eos (Washington DC)* **82**, (2001).

[22] Syvitski J (2022). Earth’s sediment cycle during the Anthropocene. Nature Reviews Earth & Environment 2022 3:3.

[23] García-Ruiz JM (2015). A meta-analysis of soil erosion rates across the world. Geomorphology.

[24] Maetens, W. *et al*. Effects of land use on annual runoff and soil loss in Europe and the Mediterranean: A meta-analysis of plot data. **36**, 599–653, 10.1177/0309133312451303 (2012).

[25] Vanmaercke M, Poesen J, Verstraeten G, de Vente J, Ocakoglu F (2011). Sediment yield in Europe: Spatial patterns and scale dependency. Geomorphology.

[26] Gonzalez-Hidalgo JC, de Luis M, Batalla RJ (2009). Effects of the largest daily events on total soil erosion by rainwater. An analysis of the USLE database. Earth Surf Process Landf.

[27] Gonzalez-Hidalgo JC, Batalla RJ, Cerda A (2013). Catchment size and contribution of the largest daily events to suspended sediment load on a continental scale. Catena (Amst).

[28] Vercruysse K, Grabowski RC, Rickson RJ (2017). Suspended sediment transport dynamics in rivers: Multi-scale drivers of temporal variation. Earth Sci Rev.

[29] Vereecken H (2015). Soil hydrology: Recent methodological advances, challenges, and perspectives. Water Resour Res.

[30] Bogena HR (2018). Toward Better Understanding of Terrestrial Processes through Long-Term Hydrological Observatories. Vadose Zone Journal.

[31] Lefèvre C, Cruse RM, Cunha dos Anjos LH, Calzolari C, Haregeweyn N (2020). Guest editorial – soil erosion assessment, tools and data: A special issue from the Global Symposium on soil Erosion 2019. International Soil and Water Conservation Research.

[32] Latron J, Lana-Renault N (2018). The relevance of hydrological research in small catchments- a perspective from long-term monitoring sites in. Europe. Geographical Research Letters.

[33] Brazier, R. E., Beven, K. J., Freer, J. & Rowan, J. S. Equifinality and uncertainty in physically based soil erosion models: application of the GLUE methodology to WEPP–the Water Erosion Prediction Project–for sites in the UK and USA. 10.1002/1096-9837.

[34] Beven K, Binley A (1992). The future of distributed models: Model calibration and uncertainty prediction. Hydrol Process.

[35] van Oost K (2005). Spatially distributed data for erosion model calibration and validation: The Ganspoel and Kinderveld datasets. Catena (Amst).

[36] Onnen N (2019). Distributed water erosion modelling at fine spatial resolution across Denmark. Geomorphology.

[37] Alatorre LC, Beguería S, García-Ruiz JM (2010). Regional scale modeling of hillslope sediment delivery: A case study in the Barasona Reservoir watershed (Spain) using WATEM/SEDEM. J Hydrol (Amst).

[38] Jetten V, de Roo A, Favis-Mortlock D (1999). Evaluation of field-scale and catchment-scale soil erosion models. Catena (Amst).

[39] Refsgaard JC (1997). Parameterisation, calibration and validation of distributed hydrological models. J Hydrol (Amst).

[40] Baartman JEM, Jetten VG, Ritsema CJ, de Vente J (2012). Exploring effects of rainfall intensity and duration on soil erosion at the catchment scale using openLISEM: Prado catchment, SE Spain. Hydrol Process.

[41] de Vente J, Poesen J (2005). Predicting soil erosion and sediment yield at the basin scale: Scale issues and semi-quantitative models. Earth Sci Rev.

[42] Batista PVG, Davies J, Silva MLN, Quinton JN (2019). On the evaluation of soil erosion models: Are we doing enough?. Earth Sci Rev.

[43] Harmel RD, Bonta JV, Richardson CW (2007). The Original USDA-ARS Experimental Watersheds in Texas and Ohio: Contributions from the Past and Visions for the Future. Trans ASABE.

[44] Owens LB, Bonta JV, Shipitalo MJ (2010). USDA-ARS North Appalachian Experimental Watershed: 70-Year Hydrologic, Soil Erosion, and Water Quality Database. Soil Science Society of America Journal.

[45] Goodrich DC (2020). The USDA‐ARS Experimental Watershed Network – Evolution, Lessons Learned, Societal Benefits, and Moving Forward. Water Resour Res.

[46] Nearing MA, Foster GR, Lane LJ, Finkner SC (1989). A Process-Based Soil Erosion Model for USDA-Water Erosion Prediction Project Technology. Transactions of the ASAE.

[47] Vines TH (2014). The Availability of Research Data Declines Rapidly with Article Age. Current Biology.

[48] Panagos P (2022). Soil priorities in the European Union. Geoderma Regional.

[49] Horowitz AJ, Clarke RT, Merten GH (2015). The effects of sample scheduling and sample numbers on estimates of the annual fluxes of suspended sediment in fluvial systems. Hydrol Process.

[50] Horowitz AJ (2003). An evaluation of sediment rating curves for estimating suspended sediment concentrations for subsequent flux calculations. Hydrol Process.

[51] Asselman NEM (2000). Fitting and interpretation of sediment rating curves. J Hydrol (Amst).

[52] Navratil O (2011). Global uncertainty analysis of suspended sediment monitoring using turbidimeter in a small mountainous river catchment. J Hydrol (Amst).

[53] Rode M, Suhr U (2007). Uncertainties in selected river water quality data. Hydrol Earth Syst Sci.

[54] Skarbøvik E, Stålnacke P, Bogen J, Bønsnes TE (2012). Impact of sampling frequency on mean concentrations and estimated loads of suspended sediment in a Norwegian river: Implications for water management. Science of The Total Environment.

[55] de Girolamo AM, di Pillo R (2018). lo Porto, A., Todisco, M. T. & Barca, E. Identifying a reliable method for estimating suspended sediment load in a temporary river system. Catena (Amst).

[56] Thodsen H (2019). Suspended matter and associated contaminants in Danish streams: a national analysis. J Soils Sediments.

[57] Gonzalez-Hidalgo JC, Batalla RJ, Cerdá A, de Luis M (2010). Contribution of the largest events to suspended sediment transport across the USA. Land Degrad Dev.

[58] Gonzalez-Hidalgo JC, Peña-Monné JL, de Luis M (2007). A review of daily soil erosion in Western Mediterranean areas. Catena (Amst).

[59] Parsons AJ, Brazier RE, Wainwright J, Powell DM (2006). Scale relationships in hillslope runoff and erosion. Earth Surf Process Landf.

[60] Kirkby MJ (2010). Distance, time and scale in soil erosion processes. Earth Surf Process Landf.

[61] Cerdan O (2004). Scale effect on runoff from experimental plots to catchments in agricultural areas in Normandy. J Hydrol (Amst).

[62] Peña-Angulo D (2019). Spatial variability of the relationships of runoff and sediment yield with weather types throughout the Mediterranean basin. J Hydrol (Amst).

[63] Panagos P (2022). European Soil Data Centre 2.0: Soil data and knowledge in support of the EU policies. Eur J Soil Sci.

[64] Matthews F (2023). Figshare.

[65] Cantreul V, Pineux N, Swerts G, Bielders C, Degré A (2020). Performance of the LandSoil expert-based model to map erosion and sedimentation: application to a cultivated catchment in central Belgium. Earth Surf Process Landf.

[66] Pineux N (2017). Diachronic soil surveys: A method for quantifying long-term diffuse erosion?. Geoderma Regional.

[67] Steegen A (2000). Sediment export by water from an agricultural catchment in the Loam Belt of central Belgium. Geomorphology.

[68] Steegen A (2001). Factors Controlling Sediment and Phosphorus Export from Two Belgian Agricultural Catchments. J Environ Qual.

[69] Steegen A, Govers G (2001). Correction factors for estimating suspended sediment export from loess catchments. Earth Surf Process Landf.

[70] Pak, L. T. *et al*. Observatoire Pesticeros des transferts de substances actives phytosanitaires dans les eaux de ruissellement d’un bassin versant agricole représentatif des régions limoneuses en grandes cultures. in *48th congress of French pesticides Group* (2018).

[71] Ouvry, J.-F. *et al*. Erosion des sols à l’échelle du bassin versant agricole de Bourville. in *Journée d’Etudes des Sols* (‘Journée d’Etudes des Sols’ conference, 2018).

[72] Grangeon T (2020). Les observatoires du ruissellement: comprendre les processus pour améliorer les modélisations. La Houille Blanche - Revue internationale de l’eau.

[73] Grangeon T (2022). Dynamic parameterization of soil surface characteristics for hydrological models in agricultural catchments. Catena (Amst).

[74] Richet, J.-B., Ouvry, J.-F. & Pak, L. T. Quantification des ruissellements sur les petits bassins versants limoneux et karstiques de Normandie. in *SHF scientific research congress Lyon 30 nov-2 déc 2020* (SHF scientific research congress Lyon 30 nov-2 déc 2020, 2020).

[75] Patault E, Alary C, Franke C, Gauthier A, Abriak NE (2019). Assessing temporal variability and controlling factors of the sediment budget of a small agricultural catchment in Northern France (the Pommeroye). Heliyon.

[76] Licciardello F, Barbagallo S, Gallart F (2019). Hydrological and erosional response of a small catchment in Sicily. Journal of Hydrology and Hydromechanics.

[77] Carollo FG, di Stefano C, Ferro V, Pampalone V (2016). New Stage-Discharge Equation for the SMBF Flume. Journal of Irrigation and Drainage Engineering.

[78] Ferro V, di Stefano C, Giordano G, Rizzo S (1998). Sediment delivery processes and the spatial distribution of caesium-137 in a small Sicilian basin. Hydrol Process.

[79] Zumr D, Dostál T, Devátý J (2015). Identification of prevailing storm runoff generation mechanisms in an intensively cultivated catchment. J. Hydrol. Hydromech.

[80] Zumr D (2017). Experimental determination of the flood wave transformation and the sediment resuspension in a small regulated stream in an agricultural catchment. Hydrol Earth Syst Sci.

[81] Li T, Jeřábek J, Noreika N, Dostál T, Zumr D (2021). An overview of hydrometeorological datasets from a small agricultural catchment (Nučice) in the Czech Republic. Hydrol Process.

[82] Gamvroudis C, Nikolaidis NP, Tzoraki O, Papadoulakis V, Karalemas N (2015). Water and sediment transport modeling of a large temporary river basin in Greece. Science of The Total Environment.

[83] Tzoraki, O. *et al*. Flood generation and classification of a semi-arid intermittent flow watershed: Evrotas river. **11**, 77–92, 10.1080/15715124.2013.768623 (2013).

[84] Smolska, E. Soil erosion and sediment supply to a fluvial system in the last-glacial area on the example of the upper Szeszupa river catchment (NE Poland). in *Zeitschrift für Geomorphologie* (2012).

[85] Smolska, E. Soil erosion and fluvial transport monitoring in the Upper Szeszupa catchment (NE Poland). in *Quaestiones Geographicae* 73–83 (Adam Mickiewicz University Press, 2008).

[86] Smolska, E. Extreme rainfalls and their impact on slopes based on soil erosion measurements (as exemplified by the Suwalki Lakeland, Poland). *Geogr Pol* **80** (2007).

[87] Smolska, E. Channel response to flood flows on example of the Szeszupa river in the last-glacial area (NE Poland). in *Quaestiones Geographicae* 63–72 (Adam Mickiewicz University Press, 2008).

[88] Święchowicz J (2002). Linkage of slope wash and sediment and solute export from a foothill catchment in the Carpathian Foothills of South Poland. Earth Surf Process Landf.

[89] Święchowicz J (2002). The influence of plant cover and land use on slope–channel decoupling in a foothill catchment: a case study from the Carpathian Foothills, southern Poland. Earth Surf Process Landf.

[90] Nunes JP (2018). Hydrological and Erosion Processes in Terraced Fields: Observations from a Humid Mediterranean Region in Northern Portugal. Land Degrad Dev.

[91] Nunes JP (2020). Impacts of wildfire and post-fire land management on hydrological and sediment processes in a humid Mediterranean headwater catchment. Hydrol Process.

[92] Wu J, Baartman JEM, Nunes JP (2021). Comparing the impacts of wildfire and meteorological variability on hydrological and erosion responses in a Mediterranean catchment. Land Degrad Dev.

[93] Bezak N, Šraj M, Mikoš M (2016). Analyses of suspended sediment loads in Slovenian rivers. Hydrological Sciences Journal.

[94] Durán ZVH (2012). Runoff and sediment yield from a small watershed in southeastern Spain (Lanjarón): implications for water quality. Hydrological Sciences Journal.

[95] Merchán D (2019). Dissolved solids and suspended sediment dynamics from five small agricultural watersheds in Navarre, Spain: A 10-year study. Catena (Amst).

[96] Casalí J (2008). Runoff, erosion, and water quality of agricultural watersheds in central Navarre (Spain). Agric Water Manag.

[97] Merchán D (2018). Runoff, nutrients, sediment and salt yields in an irrigated watershed in southern Navarre (Spain). Agric Water Manag.

[98] Chahor Y (2014). Evaluation of the AnnAGNPS model for predicting runoff and sediment yield in a small Mediterranean agricultural watershed in Navarre (Spain). Agric Water Manag.

[99] Giménez R (2012). Factors controlling sediment export in a small agricultural watershed in Navarre (Spain). Agric Water Manag.

[100] Casalí J (2010). Sediment production and water quality of watersheds with contrasting land use in Navarre (Spain). Agric Water Manag.

[101] Outeiro L, Úbeda X, Farguell J (2010). The impact of agriculture on solute and suspended sediment load on a Mediterranean watershed after intense rainstorms. Earth Surf Process Landf.

[102] Farguell J, Úbeda X, Pacheco E (2022). Shrub removal effects on runoff and sediment transport in a mediterranean experimental catchment (Vernegà River, NE Spain). Catena (Amst).

[103] Fick SE, Hijmans RJ (2017). WorldClim 2: new 1-km spatial resolution climate surfaces for global land areas. International Journal of Climatology.

[104] Metzger MJ, Bunce RGH, Jongman RHG, Mücher CA, Watkins JW (2005). A climatic stratification of the environment of Europe. Global Ecology and Biogeography.

